# Heterozygous Mutations in Both the *AMN* and *CBS* Genes: Double Haploinsufficiency as an Unusual Cause of Vitamin B_12_ Deficiency—A Case Report

**DOI:** 10.1002/ccr3.9600

**Published:** 2024-11-22

**Authors:** Per Ole Iversen, Jean‐Louis Gueant, Abderrahim Oussalah, Helga Refsum, Ebba Nexo, Geir E. Tjønnfjord, Christian Qvigstad

**Affiliations:** ^1^ Department of Nutrition University of Oslo Oslo Norway; ^2^ Department of Haematology Oslo University Hospital Oslo Norway; ^3^ Department of Molecular Medicine, Division of Biochemistry, Molecular Biology, and Nutrition University Hospital of Nancy and INSERM UMR_S 1256, Faculty of Medicine of Nancy Nancy France; ^4^ Department of Clinical Medicine/Biochemistry Aarhus University Hospital Aarhus Denmark; ^5^ Institute of Clinical Medicine University of Oslo Oslo Norway

**Keywords:** folic acid, general medicine, hematology, vitamin B12

## Abstract

Vitamin B_12_ deficiency is usually simple to diagnose. However, our patient demonstrates that in difficult cases, the ordinary clinician may need a transdisciplinary approach. The finding of a double haploinsufficiency as a possible cause of vitamin B_12_ deficiency in our patient, illustrates the usefulness of performing large panel clinical exome sequencing.

## Graphical abstract

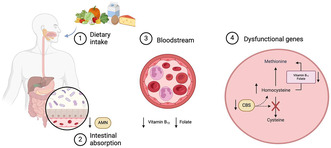

## 1 Introduction

Common causes of vitamin B_12_ deficiency are inadequate dietary intake of food containing vitamin B_12_ (e.g., among undernourished, the elderly, vegetarians/vegans, drug addicts, or among subjects with increased needs such as in pregnancy), and digestive diseases with malabsorption (e.g., atrophic gastritis, inflammatory bowel diseases such as Crohn's disease, celiac disease, bariatric surgery or surgical resection of part of the gut) [1]. Other causes are iatrogenic‐induced malabsorption (e.g., treatments with proton pump inhibitors and metformin) and less frequently inherited diseases affecting the transport and metabolism of vitamin B_12_. Vitamin B_12_ deficiency may also produce intestinal mucosal atrophy with subsequent folate malabsorption [2]. Folate deficiencies are mostly related to insufficient dietary intake. Notably, no specific defect in proteins related to the uptake or trafficking of folate has been related to low levels of plasma folate [3]. Vitamin B_12_ deficiency may lead to a spuriously increased plasma folate level that will decline upon treatment with vitamin B_12_ [4]. We report here a young woman who presented initially with a combined vitamin B_12_ and folate deficiency of unknown cause.

## 2 Case History/Examination

In 2012, a 22‐year‐old Caucasian nulliparous woman was referred by her general practitioner (GP) to her local hospital due to symptoms and signs of anemia over the past few months. She was otherwise healthy, was not on any restricted diet, had never undergone surgery, and used no medication regularly except Yasmin (birth control pill containing estrogen and progesterone). A clinical examination revealed no abnormalities. Analyses of a blood sample (see Table 1 for reference values) showed a low hemoglobin (Hb) level of 6.9 g/dL, a sub‐normal platelet count of 110 × 10^9^/L, a mean corpuscular volume (MCV) of 127 fL, reticulocytes 50 × 10^9^/L, and a white blood count (WBC) of 5.5 × 10^9^/L, including a normal differential count. A blood smear disclosed macrocytosis, hyper‐segmented granulocytes, and no signs of blast cells. Plasma ferritin was 217 μg/L with a transferrin saturation of 58% and undetectable haptoglobin. Vitamin B_12_ was low (50 pmol/L) and folate level was subnormal (6 nmol/L). Na, K, creatinine, bilirubin, aspartate aminotransferase, alanine aminotransferase, and C‐reactive protein were all within the reference values.

**TABLE 1:** Reference ranges for blood levels of the measured compounds.

Compound^a^	Reference range	Unit
Hemoglobin	11.7–15.3	g/dL
Platelets	145–390	× 10^9^/L
White blood cells	3.5–10.0	× 10^9^/L
MCV	82–98	fL
Iron saturation	10–50	%
Ferritin	10–170	μg/L
Vitamin B_12_	150–650	pmol/L
Folate	> 7	nmol/L
Homocysteine	5.0–15.0	μmol/L
Methylmalonic acid	< 0.30	μmol/L
Haptoglobin	0.4–2.1	g/L
Lactate dehydrogenase	105–205	U/L
Transcobalamin	560–1550	pmol/L
Haptocorrin	240–680	pmol/L
Vitamin B_6_	15–160	nmol/L

^a^ Reference values are from Oslo University Hospital, Oslo, Norway; except for transcobalamin and haptocorrin as they are from Aarhus University Hospital, Aarhus, Denmark.

The patient was discharged with a possible diagnosis of vitamin B_12_ deficiency and started with vitamin B_12_ injections (1 mg im) weekly for 5 weeks, then planned for every 3 months, as per our national guidelines. She was readmitted 3 days later for a scheduled bone marrow examination and an upper endoscopy. Her blood tests were virtually unchanged. Due to her low blood levels of vitamin B_12_ and Hb and characteristic findings on the blood smear, vitamin B_12_ deficiency was assumed to be the most likely diagnosis, and the planned bone marrow examination was therefore not performed. Gastric biopsies taken during upper endoscopy showed no pathological findings, in particular, no antro‐fundic atrophy and no duodenal villous atrophy. No anti‐intrinsic factor, parietal cell, or tissue transglutaminase antibodies were detected. Analyses of fecal samples (including an elastase test) did not reveal pancreas dysfunction or signs of an inflammatory bowel disease. She was discharged with the initially suggested treatment with vitamin B_12_ injections.

The patient was well for the next few years, and she had no encounters with her GP or the local hospital. It is unclear how long she continued with the vitamin B_12_ injections.

About 3.5 years (in 2016) later, she was admitted to the labor ward at Oslo University Hospital at gestational week 40^+1^. Earlier during the otherwise normal pregnancy, she was again diagnosed with macrocytic anemia (Hb 7.2 g/dL, MCV 102 fL) and combined vitamin B_12_ (80 pmol/L) and folate (4.5 nmol/L) deficiency. On the day of admission, she received two units of packed red cells, and 2 weeks later, Hb was 8.1 g/dL. A healthy boy (birth weight 2858 g) was born 2 days later by vaginal delivery with a maternal blood loss of 1 L. She was discharged after 5 days and advised to take vitamin B_12_ injections (1 mg im) every 3 months and oral folic acid (1 mg daily).

In the following 5 years, the patient had no specific health challenges. She worked as a nurse. She has three siblings, of whom one brother reportedly suffered from chronic anemia of unknown cause. Following an outpatient evaluation, he had normal laboratory values and no clinical signs of anemia. We have no other information about her family or any inherited diseases therein. Specifically, the patient was unaware of any relatives with vitamin B_12_ deficiency, folate deficiency, or other blood‐related diseases.

The next contact with the health service was in 2021, when she was referred to our outpatient clinic due to several blistering wounds/shingles in the mouth, along the base of the tongue and in the gingiva. On suspicion of a viral infection, her GP had started oral valacyclovir 500 mg BD for 2 months. Assessment of biopsy specimens from affected areas could not confirm a viral etiology. Hence, the antiviral medication was discontinued, and the oral lesions eventually disappeared. However, she remained fatigued and fainted at the GP's office before being transferred to the Department of Haematology, Oslo University Hospital. At that time, her Hb was 3.5 g/dL, MCV 115 fL, WBC 2.9 × 10^9^/L, and platelet count 72 × 10^9^/L. Furthermore, vitamin B_12_ was 70 pmol/L, folate < 5 nmol/L, homocysteine 71 μmol/L, methylmalonic acid 0.22 μmol/L and haptoglobin undetectable whereas lactate dehydrogenase was 1936 U/L, indicating hemolysis. She had lost 15 kg of body weight during the previous 2 months. An extensive serological examination was performed, but the analyses were negative for human immunodeficiency virus, varicella‐zoster virus, Epstein–Barr virus, cytomegalovirus, hepatitis B, and C virus, treponema pallidum and toxoplasma gondii. A bone marrow examination was consistent with vitamin B_12_ and/or folate deficiency. Gastro‐duodenoscopy was normal, and there was no added information from other investigations including negative findings regarding antibodies toward intrinsic factor, parietal cells, tissue transglutaminase, and deamidated‐gliadin peptide.

During this hospital stay, she received a 4‐day treatment with injections of vitamin B_12_ (1 mg im daily) and two units of packed red blood cells. She was discharged and encouraged to use oral vitamin B_12_ (since her vitamin B_12_ stores probably had been filled with the intramuscular injections), 2 mg daily the first month and then 1 mg in addition to 5 mg of folic acid daily. At the time of discharge, most of her blood values normalized. On treatment with oral vitamin B_12_ and folate, her vitamin B_12_ levels decreased slowly with time, from 1380 pmol/L at discharge to 318 pmol/L (i.e., still within the reference range) while folate remained > 20 nmol/L 1 year later (March 2022). Due to the gradual decrease in vitamin B_12_ levels, we converted the vitamin B_12_ treatment from oral supplements to injections (1 mg im every 3 months). As it was somewhat unclear if the low folate levels could initially have caused the diagnosis of vitamin B_12_ deficiency, we stopped the vitamin B_12_ treatment in December 2022 and kept her only on folate supplementation. Whereas the vitamin B_12_ levels continued to decrease (to about 260 pmol/L in February 2024), she maintained adequate folate levels, indicating that folate deficiency was probably not the cause of her vitamin B_12_ deficiency. During the same period, homocysteine decreased but remained slightly elevated (18–23 μmol/L), while methylmalonic acid was within the reference range (< 0.30 μmol/L). We then (February 2024) decided to resume treatment with injections of vitamin B_12_ (1 mg im every 3 months).

## 3 Differential Diagnosis, Investigations, and Treatment

Since the most common causes of vitamin B_12_ deficiency were ruled out (i.e., dietary insufficiency, atrophic gastritis, and iatrogenic causes), we next studied more sophisticated causes. On suspicion of a defect in the transport or the intestinal absorption of vitamin B_12_, we measured the levels of transcobalamin and haptocorrin (1380 and 529 pmol/L, respectively), two compounds important for vitamin B_12_ transport. They were, however, within the reference range. Next, we explored vitamin B_12_ uptake using the CobaSorb test [5]. We found an increase of 37% in vitamin B_12_ bound to transcobalamin, compatible with a normal uptake of vitamin B_12_ from the administered test dose of 3 times 9 μg of cyanocobalamin for 2 days.

**FIGURE 1:**
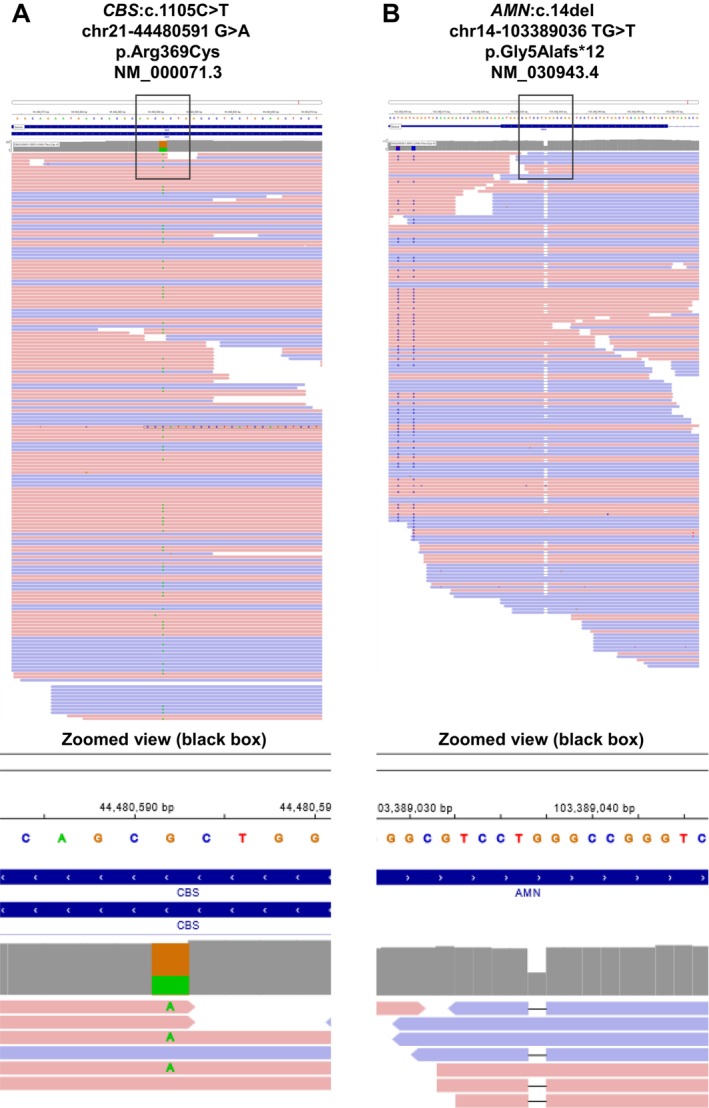
Genomic context of the *CBS* (CBS: C.1105C>T) (A) and *AMN* (AMN: C.14del) (B) variants identified in the patient. A zoomed view (indicated by the black box) displays an enlarged and detailed image, enhancing visualization of the area of interest.

A consistent finding in our patient during the period we investigated her was elevated levels of homocysteine concurrent with normal levels of methylmalonic acid. We therefore screened for possible abnormal levels of one‐carbon metabolites and mutations of genes involved in vitamin B_12_‐ and folate metabolism. The serum metabolomic analysis of markers of one‐carbon metabolism showed an increase in choline, a marked decrease in betaine, and a decrease in dimethylglycine. These findings were consistent with a metabolic block that impaired the conversion of choline into betaine by choline dehydrogenase. We further performed clinical exome sequencing [6] to examine the genes involved in vitamin B_12_ absorption, including the cobalamin gastric intrinsic factor gene (*CBLIF*), the amnion‐associated transmembrane gene (*AMN*) gene, and the cubilin gene (*CUBN*) which encode the two proteins of the intestinal receptor complex necessary for the absorption of the vitamin B_12_‐intrinsic factor complex in the distal ileum [2]. Interestingly, we could detect a heterozygous mutation in the *AMN* gene; AMN is the transmembrane component of the cubam intrinsic factor receptor expressed in the ileum. Surprisingly, in addition to the *AMN* heterozygous mutation, we found a heterozygous mutation in the cystathionine beta‐synthase gene (*CBS*) which encodes the enzyme catalyzing the formation of cystathionine from homocysteine and serine with pyridoxal phosphate (i.e., vitamin B_6_) as a cofactor (Figure 1). Notably, the level of vitamin B_6_ (32 nmol/L) in our patient was within the reference range.

## 4 Outcome and Follow‐Up

Homozygous mutation in the *AMN* gene is a genetic defect causing the Imerslund–Gräsbeck disease (IGS), leading to impaired intestinal absorption of vitamin B_12_ [1]. Our patient had a known Norwegian founder mutation [7]. The occurrence of IGS in Norway is 1:200,000, and the carrier frequency is 1:224. Notably, about half of the patients with IGS have proteinuria due to mutations in *CUBN* [1], which was not the case in our patient. A decreased activity of the CBS enzyme, for example, due to haploinsufficiency in our patient with a heterozygous mutation in *CBS*, may explain the persistent slightly increased homocysteine levels despite recovery of normal vitamin B_12_‐ and folate levels. Our patient is now substituted with vitamin B_12_ injections (1 mg im every 3 months) and oral folic acid (1 mg daily). She is doing well with no clinical manifestations of vitamin B_12_ or folate deficiency.

## 5 Discussion

In most patients diagnosed with vitamin B_12_ deficiency, the cause of the disorder is straightforward to identify [1]. Our patient demonstrates two important issues. One is the challenge an ordinary clinician may face when searching for the cause of vitamin B_12_ deficiency is unclear. As in our case, the involvement of colleagues with different skills supports a transdisciplinary approach. The other issue relates to the finding of the double haploinsufficiency, illustrating the usefulness of performing clinical exome sequencing, as assessment of metabolomics was not particularly helpful in our case.

The *AMN* and *CBS* gene mutations probably led to a combined haploinsufficiency. To the best of our knowledge, this abnormality has not been reported previously. *AMN* encodes the amnionless protein, part of the cubam heteromeric complex that ensures the uptake and endocytosis of the intrinsic factor‐vitamin B_12_ complex in the distal ileum [8]. Whereas homozygous mutation in the *AMN* gene is the genetic defect in IGS [1], the role of heterozygous mutations in this gene for vitamin B_12_ intestinal absorption has apparently not been previously explored. However, our present data on intestinal vitamin B_12_ uptake suggests that a heterozygous mutation in *AMN* does not influence intestinal vitamin B_12_ absorption.

The heterozygous mutation in the *CBS* gene could have influenced the metabolic presentation of our patient through haploinsufficiency, as homocysteine remained at a slightly higher level after vitamin B_12_ and folate substitution. The decrease of the transsulfuration pathway was limited since the metabolomic study showed no decrease in cystathionin and cysteine concentrations. The decrease of betaine and increase of choline levels could be related to the increase of the betaine/choline‐dependent remethylation catalyzed by betaine homocysteine methyltransferase, which plays a compensatory role when the vitamin B_12_‐dependent remethylation pathway is impaired. However, in the absence of vitamin B_6_ and vitamin B_12_ deficiencies, the metabolic consequence was limited and had most likely no consequence on treatment efficacy.

## 6 Conclusion

**FIGURE 2:**
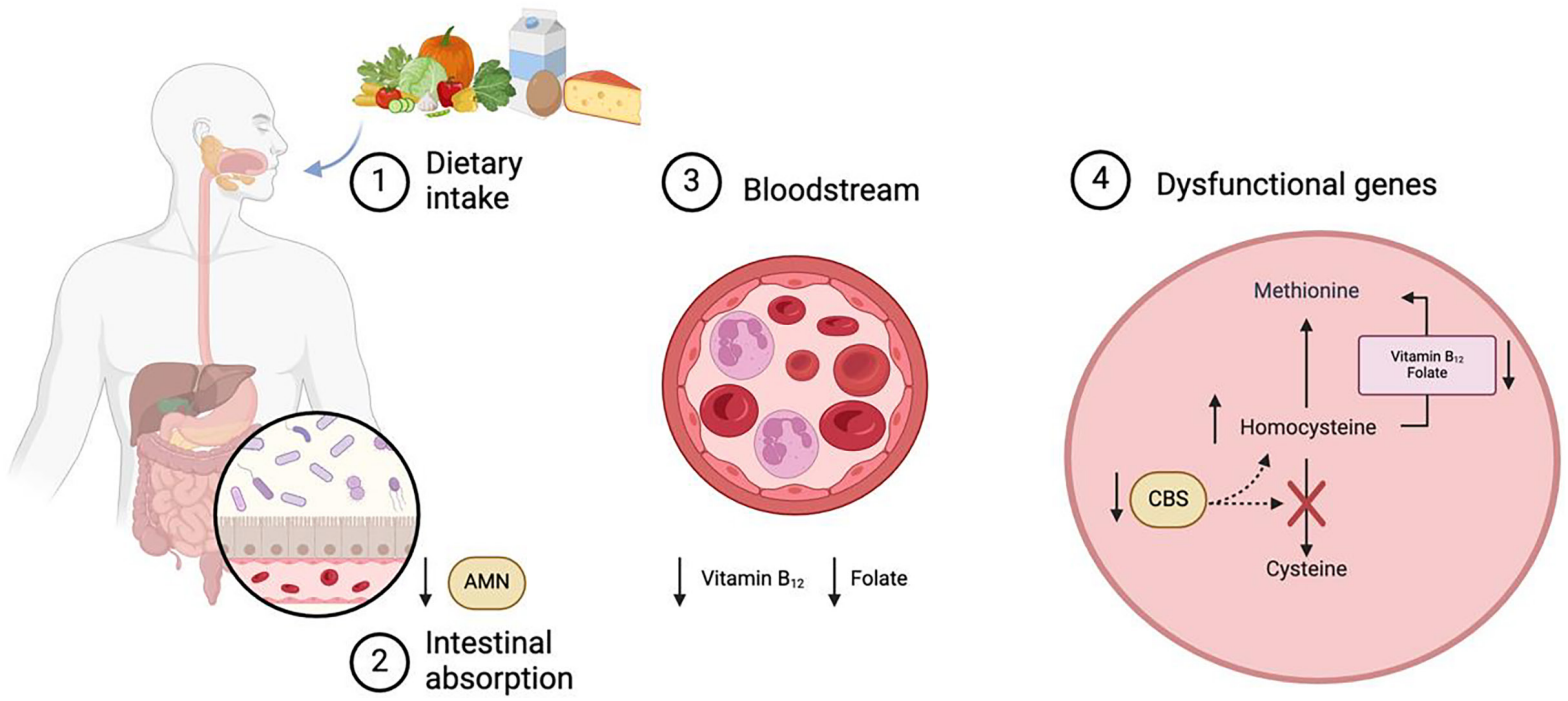
This schematic figure (created with BioRender.com) illustrates the key steps involved in the disturbed metabolic pathway that may explain the low vitamin B_12_ and folate levels in our patient: (1) Dietary intake: The primary source for vitamin B_12_ and folate is through dietary consumption of protein‐rich foods and vegetables. (2) Intestinal absorption: Vitamin B_12_ and folate are primarily absorbed from the small intestine into the bloodstream. (3) Bloodstream: Homocysteine, vitamin B_12_ and folate levels in the bloodstream are influenced by the balance between uptake/production and removal. (4) Dysfunctional genes: Defects in two key enzymes encoded by the *CBS* and the *AMN* genes can lead to both impaired homocysteine metabolism and vitamin B_12_ malabsorption.

In conclusion, we have described a female patient with a combined vitamin B_12_ and folate deficiency, housing heterozygous mutations in both the *AMN* and *CBS* genes (Figure 2). A prolonged investigation with progressive involvements of various medical sub‐specialties was needed to unravel this rare case. Furthermore, this case illustrates that unraveling unexplained vitamin B_12_ deficiency may be challenging, and it may need a multidisciplinary approach. These cases are most likely very rear, but dissecting the detailed pathophysiology of unexplained vitamin B_12_ deficiency may result in improved patient care and it deepens our understanding of vitamin B_12_ biology. Further studies are warranted to scrutinize a possible causal relationship between the double haploinsufficiency and the clinical picture.

## Author Contributions

**Per Ole Iversen:** conceptualization, investigation, methodology, project administration, writing – original draft, writing – review and editing. **Jean‐Louis Gueant:** formal analysis, investigation, writing – review and editing. **Abderrahim Oussalah:** data curation, formal analysis, writing – review and editing. **Helga Refsum:** conceptualization, writing – review and editing. **Ebba Nexo:** formal analysis, investigation, writing – review and editing. **Geir E. Tjønnfjord:** conceptualization, investigation, writing – review and editing. **Christian Qvigstad:** conceptualization, investigation, writing – review and editing.

## Consent

Written consent has been obtained from the patient to publish this report in accordance with the journal's patient consent policy.

## Conflicts of Interest

The authors declare no conflicts of interest.

## Data Availability

As this article details medical information about one individual, data will not be made available.
